# Assessment of the Feasibility of Using Additive Manufacturing from Metal Powder to Produce Compact Heat Exchangers

**DOI:** 10.3390/ma18133035

**Published:** 2025-06-26

**Authors:** Katarzyna Chliszcz, Dorota Laskowska, Waldemar Kuczyński, Błażej Bałasz, Maciej Kasperowaicz, Kevin Moj

**Affiliations:** 1 Faculty of Mechanical Engineering and Energy, Koszalin University of Technology, Śniadeckich 2, 75-453 Koszalin, Poland; waldemar.kuczynski@tu.koszalin.pl (W.K.); blazej.balasz@tu.koszalin.pl (B.B.); maciej.kasperowicz@tu.koszalin.pl (M.K.); 2 Faculty of Mechanical Engineering, Opole University of Technology, ul. Proszkowska 76, 45-758 Opole, Poland; ke.moj@po.edu.pl

**Keywords:** additive manufacturing from metal powders, compact heat exchangers, minichannels, stainless steel 316L

## Abstract

The miniaturization of heat exchangers requires advanced manufacturing methods, as conventional techniques such as milling or casting are insufficient for producing complex microscale geometries. This study investigates the feasibility of using selective laser melting (SLM) with 316L stainless steel powder to fabricate compact heat exchangers with minichannels. The exchanger was designed using Autodesk Inventor 2023.3 software and produced under optimized process parameters. Measurements using a hydrostatic balance demonstrated that the applied process parameters resulted in a relative material density of 99.5%. The average microhardness in the core region of the SLM-fabricated samples was 255 HV, and the chemical composition of the final material differed only slightly from that of the feedstock material (stainless steel powder). Dimensional accuracy, surface quality, and internal structure integrity were assessed using computed tomography, optical microscopy, and contact profilometry. The fabricated component demonstrated high geometric fidelity and channel permeability, with local surface deformations associated with the absence of support structures. The average surface roughness (Ra) of the minichannels was 11.11 ± 1.63 µm. The results confirm that SLM technology enables the production of functionally viable heat exchangers with complex geometries. However, limitations remain regarding dimensional accuracy, powder removal, and surface roughness. These findings highlight the potential of metal additive manufacturing for heat transfer applications while emphasizing the need for further research on performance testing under real operating conditions, especially involving two-phase flow.

## 1. Introduction

Heat exchangers are technical devices that are used to intentionally transfer thermal energy between two separate fluids of different temperatures. They play a significant role in virtually all industries, in particular power generation, electronics [1,2,3], automotive [4], space technology, construction, power systems, etc. [5]. Such a wide range of applications makes it necessary to constantly improve and innovate them to increase heat transfer efficiency [6]. Due to current regulatory requirements, one of the key criteria in selecting a refrigerant as a working fluid in heat exchangers is its environmental impact. The widely used R134a is being gradually phased out because of its high global warming potential. It is being replaced by newer refrigerants such as R1234yf and R1234ze(E), which are considered more environmentally friendly. However, their use introduces additional safety considerations, as they are classified as mildly flammable. This necessitates the design of heat exchange devices with reduced refrigerant charge. As a result, contemporary research increasingly focuses on the miniaturization of heat exchanger structures [7]. Therefore, modern research in the field of heat exchangers is largely focused on the issue of miniaturization. This is because it is necessary to design small-sized heat exchangers that will be characterized by material and energy efficiency in the manufacturing process and capable of transferring high heat flux density per unit volume [8]. This is how the concept of designing compact heat exchangers was established. Compact heat exchangers are defined by Shah as those with a heat transfer surface-to-volume ratio of more than 700 m^2^ m^−3^ [9,10]. The construction of such heat exchangers is often based on mini- and microchannels [7].

Production of compact exchangers by classical cavity methods (milling, drilling) or casting is often not possible [11,12]. In addition, the joining of exchanger components made in this way is generally completed by soldering or welding. If the connections made are not tight, the exchanger is prone to leakage of working fluid, which also has a negative impact on the environment when synthetic refrigerants are used. An alternative is additive manufacturing (AM) from metal powders, which makes it possible to create a physical component directly from a digital, three-dimensional model by building it up layer by layer [13,14,15,16,17]. In terms of heat transfer, selective laser melting (SLM) technology is increasingly being used.

The process begins with making a digital 3D model in CAD software. The finished file goes into special software, which divides the model into layers. At this stage, support structures are also generated, and printing parameters are defined (laser power, scanning speed, hatch distance, and layer height), which determine the properties of the manufactured part [18,19,20]. The file prepared in this way is implemented into the technological device for additive manufacturing [21].

**Figure 1:**
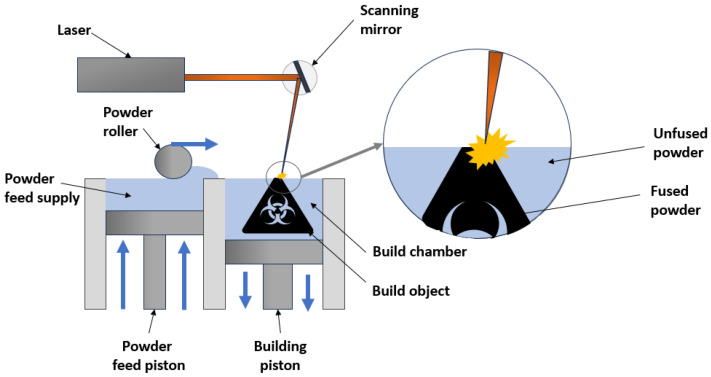
The process of creating a component using SLM technology (own elaboration based on [22]).

As shown in Figure 1, a typical device that operates with SLM technology consists of an enclosed chamber with a laser beam source with a scanning system, a tray with the construction material (metal powder), a working platform on which the part to be made is formed, and an arm (squeegee) used to distribute the powder. To prevent oxidation, the process takes place in an argon atmosphere. Fabrication begins by applying a layer of powder to the working platform. The laser beam then scans the layer according to predefined paths. After the layer is produced, the working platform is lowered, and the squeegee again applies a layer of structural material. Such a cycle is repeated until a complete part is obtained [19,20,21,22]. In a further stage, the finished part undergoes a controlled temperature reduction process. After its completion, the chamber is cleaned of unbaked powder, and the manufactured part is pulled out together with the working platform. Then, the supports attaching the part to the working platform are removed, as well as the unbaked loose powder that remains in the part. Removing the powder that is in the internal channels of the exchanger can be challenging, especially if the exchanger does not have a sufficient number of openings. This must therefore be taken into account in the design process. The final step is finishing processing, which aims to improve the surface properties of the manufactured part.

Additive technology provides opportunities that can be directly applied to heat exchanger issues. It allows the creation of complex shapes and structures, i.e., minichannels [23], cellular structures [5], and area and turbulence promoters that increase heat transfer surfaces [24,25]. Cellular materials, due to their internal porosity, offer improved structural and functional performance over solid-phase materials. Recent studies increasingly explore biologically inspired structures known as triply periodic minimal surfaces (TPMS), which are defined by trigonometric functions and characterized by zero-mean curvature and a high surface area-to-volume ratio [26]. These features make TPMS highly suitable for heat exchange applications due to enhanced surface density, low weight, and high mechanical strength. Since traditional manufacturing is inadequate for producing such complex geometries, additive manufacturing becomes essential. For instance, Dixit et al. [27] designed and fabricated a gyroid-based heat exchanger with a surface-to-volume ratio of 670 m^2^/m^−3^.

Turbulence promoters are commonly used to increase the effective heat transfer surface area, thereby enhancing the thermal performance of heat exchangers. Their primary role is to disrupt the laminar boundary layer that forms along the internal walls of the flow passages, which constitutes a major resistance to heat transfer. This approach is widely recognized as an effective and low-cost method for improving heat transfer efficiency. While both surface area enhancements and turbulence promoters can be manufactured using conventional techniques, additive manufacturing (AM) offers significant advantages in this regard. AM enables the fabrication of highly complex geometries that are difficult or even impossible to achieve using traditional methods. Moreover, it eliminates the need for subsequent bonding or assembly processes, as these structures can be integrated directly during the printing phase. Importantly, AM also facilitates the production of such features at the microscale, further supporting efforts toward miniaturization and performance optimization. Rastan et al. [28] investigated the heat transfer performance of additively manufactured rectangular minichannel heat exchangers incorporating three different types of longitudinal vortex generators, with a smooth channel serving as the baseline configuration. Their results demonstrated that the inclusion of vortex generators could enhance convective heat transfer by up to threefold, depending on the specific geometric parameters. These findings underscore the potential of additive manufacturing for the development of advanced minichannel heat exchangers with improved thermal performance.

Additive manufacturing also makes it possible to affect surface roughness. Surface roughness can intensify heat transfer. Components produced using additive manufacturing technology are characterized by relatively high surface roughness, which is affected by factors such as material [29], powder particle size, the placement of the component in the build chamber [30], build direction [16], support for protruding elements, and build angle [31]. In this process, the so-called staircase effect occurs. Surface roughness increases with the height of the layer and the build angle. Reducing the layer height can decrease the roughness of inclined surfaces, but it will extend the production time and, consequently, increase the cost of the component.

The influence of surface roughness on heat transfer was studied by Soontarapiromsook et al. [32]. The authors investigated the effect of surface roughness of plate heat exchangers (ranging from 0.594 μm to 2.754 μm) on the condensation of refrigerant 134a. The obtained data indicated that the heat transfer coefficient for the rough surface was approximately 31–44% higher than for the smooth surface.

Ventola et al. [33] also addressed this issue. They focused on the application of artificial roughness, created through additive manufacturing, to improve heat transfer in the production of flat and finned heat sinks for electronics cooling. They prepared samples made of AlSi10Mg using DLSM technology with average roughness (Ra) of 16, 24, and 43 μm, as well as a finned sample where Ra = 22 μm and a smooth milled sample (Ra = 1 μm) as a reference. The results showed that rough surfaces increased the heat transfer coefficient by up to 73% (on average 63%) compared to the smooth surface. In the case of rough-finned surfaces, the coefficient increased by a maximum of 40% (on average 35%). Surface roughness is an important factor not only in the context of heat transfer but also in the analysis of pressure drop. Stimpson et al. [15], who conducted studies in channels with hydraulic diameters between 635 and 1261 μm, indicated that as roughness increases, the friction factor also rises, leading to a greater pressure drop in the channels.

Another advantage of additive manufacturing is the ability to produce from a variety of structural materials [11]. In SLM technology, the structural material is metal powders that meet a number of requirements regarding morphology and grain size distribution [34,35,36]. The selection of the appropriate powder should be carried out taking into account the operating conditions and the boundary parameters under which the final component will be used [20]. Powder density, thermal conductivity, and cost are also important factors. Iron alloys, such as SS316L, are often used in additive manufacturing technology due to their affordability, ability to operate at high temperatures, and resistance to corrosive environments. If low heat exchanger weight is a priority and the operating temperature is below 250 °C, an aluminum alloy may be an appropriate choice, especially in electronics cooling applications [21]. Nickel-and titanium-based alloys are often used for aerospace applications due to their suitable mechanical properties at very high temperatures [37,38]. Another material can be copper, which is considered a highly attractive material for heat transfer applications due to its excellent thermal conductivity. Recent publications have reported initial successes in the use of copper for the fabrication of heat exchangers via additive manufacturing [39]. Nevertheless, the application of this material in such processes remains limited and presents significant challenges, including low absorption of laser radiation, a tendency for delamination, and deformation (e.g., bending) during production [40,41]. These limitations indicate that the additive manufacturing of copper components is still an emerging research area that requires further systematic investigation. Additive manufacturing is considered to be a technology that makes it possible to reduce energy and material consumption in the manufacturing process [42,43]. This issue was addressed by, among others, Wippermann [43], who compared SLM technology with machining. His results show that, compared to machining, SLM technology allows for almost 20 times lower average power consumption, more than twice lower specific energy consumption, and four times less waste material.

Despite the opportunities offered by additive manufacturing in heat transfer issues, it is important to remember that this technology also has limitations. Therefore, an in-depth understanding of the technology is necessary before embarking on the design process for the heat exchanger and, in particular, its internal geometry. Challenges include cleaning the component and its internal channels of unmelted powder, particularly when the exchanger has no holes [44]. The lack of repeatability in production is also a major problem, particularly for very small geometries. Measurement deviations and wall distortions are greater the smaller the thickness of the manufactured part. This applies to both the vertical and horizontal planes [45]. The fabrication of thin walls can also pose a challenge in terms of element integrity. Insufficient layer bonding may occur during the fabrication process, or the element may have too much porosity, causing fluid to leak from the exchanger or mix with the other fluid [46]. In addition, surface roughness, while it may have its advantages, is also a potential hazard, as it can alter the hydraulic diameter of the channel or even block it completely, especially if there are significant irregularities [47]. In addition to the difficulties at the design and manufacturing stages of such an AM heat exchanger, it is also a challenge to determine its performance under experimental conditions.

An attempt to use this technology in heat exchange was made by Arie et al. [48], creating air-to-water heat exchangers for power plant cooling from stainless steel (SS17-4), titanium alloy (Ti64), and aluminum alloy (AlSi10Mg). Zhang et al. [49], on the other hand, made a collector-microchannel heat exchanger from Inconel 718 for high temperature aerospace applications. Saltzman et al. [50], in their study, compared the performance of conventionally made air-liquid cross-flow plate heat exchangers used as oil coolers in aircraft with exchangers of similar geometry made using AM from AlSi10Mg aluminum alloy. Dixit et al. [27] proposed an exchanger based on cellular structures, while Romei et al. [47], using AM, created a novel, high temperature, resistive spacecraft heat exchanger. Chekurov et al. [51] presented an additively fabricated AlSi10Mg aluminum alloy counter-current heat exchanger consisting of 144 minichannels arranged in a checkerboard pattern.

## 2. Materials and Methods

### 2.1. Compact Heat Exchangers Design

**Figure 2:**
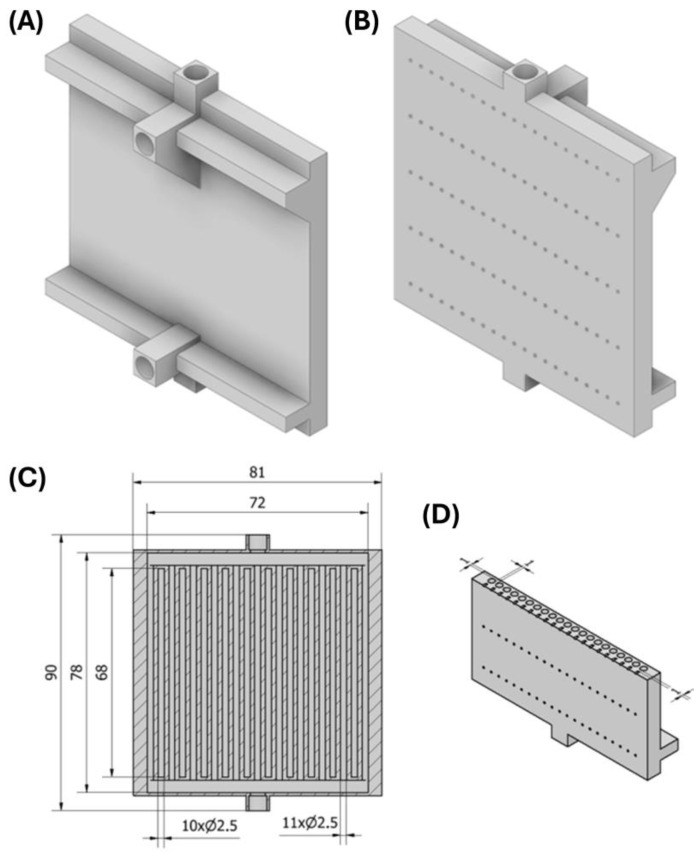
Design of a compact heat exchanger: (**A**) front 3D view, (**B**) back 3D view, (**C**) inside view with dimensions, (**D**) cross-sectional view with minichannels.

**Figure 3:**
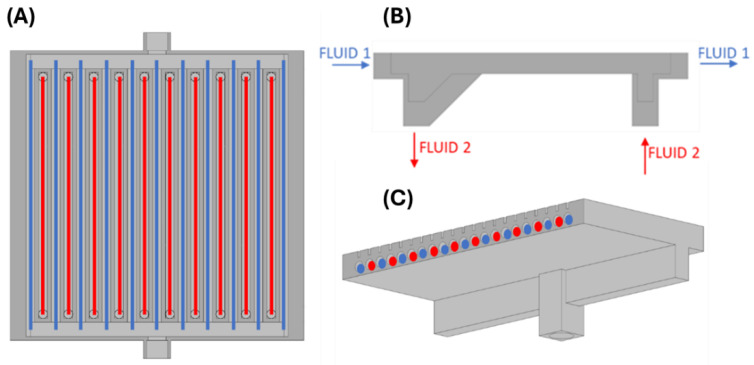
Exchanger model with determination of fluid flow: (**A**) inside view with marked loops, (**B**) side view of flow arrangement, (**C**) isometric view showing channel layout and flow. Blue color—flow of fluid 1; red color—flow of fluid 2.

Figure 2A–D show the design of a compact heat exchanger. Autodesk Inventor software 2023.3 (Autodesk, San Francisco, CA, USA) was used to design it. The total dimensions of the exchanger are 90 × 81 × 21 mm. It consists of two loops of minichannels with a circular cross-section and a hydraulic diameter of 2.5 mm each (the first loop—marked in blue—11 minichannels, the second loop—marked in red—10 minichannels) (Figure 3A–C). The minichannels of each loop are arranged alternately, parallel to one another. The wall between the minichannels is 1 mm thick. In addition, holes for thermocouples were prepared in one of the surfaces of the exchanger to be used for experimental studies.

The flow of fluids through the exchanger can be implemented in both co-current and counter-current ways. The average temperature difference in the counter-current flow is greater than in the co-current flow, which allows for transporting a larger heat flux while maintaining the same heat transfer surface and heat transfer coefficient value, or with the same heat flux, using a smaller heat transfer surface, which is particularly important from an economic point of view.

### 2.2. Compact Heat Exchanger Fabrication and Powder Characterization

**Figure 4:**
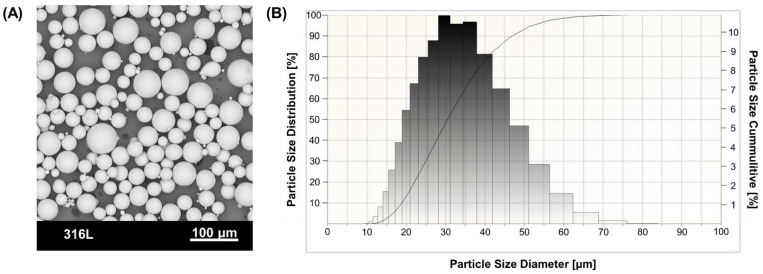
MetcoAddTM 316L-A powder characterization: (**A**) SEM morphology, (**B**) particle size distribution analysis.

The structural material was a commercially available 316L stainless steel powder with the trade name MetcoAdd^TM^ 316L-A (OerliconMetco Inc., Troy, MI, USA). Its chemical composition is shown in Table 1. Figure 4A shows the powder morphology obtained with SEM microscopy (PHENOM PRO, Thermo Fisher Inc., Waltham, MA, USA). Figure 4B shows the grain size distribution determined with a laser particle meter ANALYSETTE 22 MicroTec Plus (Fritsch GmbH, Amberg, Germany). The D90, D50, and D10 parameters characterizing the powder grain size distribution were 46 μm, 30 μm, and 19 μm, respectively.

**Figure 5:**
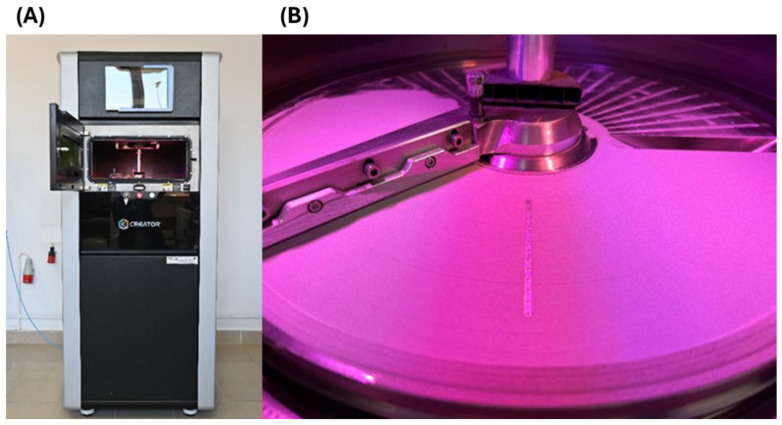
ORLAS CREATOR^®^ selective laser melting machine: (**A**) external view, (**B**) view from the working chamber.

The compact heat exchanger was manufactured using selective laser melting technology with the ORLAS CREATOR^®^ (OR LasertechnologieG, Bhm Dieburg, Germany) (Figure 5A,B). The device is equipped with a Ytterbium fiber laser, featuring a beam spot size of 40 μm, a maximum power of 250 W, and a wavelength of 1070 nm.

Table 2 shows the process parameters used. The process parameters were selected based on a previously conducted optimization experiment aimed at maximizing the relative density by varying two key process parameters: laser power and scanning speed. The study focused on cubic samples with dimensions of 10 × 10 × 10 mm, whose relative density was measured using a Mettler Toledo XS105 hydrostatic balance (Mettler Toledo, Columbus, OH, USA). For the parameter combinations presented in Table 2, a material relative density of 99.5% was achieved (with reference to the density of 316L stainless steel, approximately 8 g cm^−3^).

### 2.3. Material Characterization

Due to the specific nature of the additive manufacturing process, material characterization is important. For this purpose, cubes of dimensions 10 × 10 × 10 mm were produced according to the parameters presented in Table 2. This was necessary due to limitations of the measuring equipment.

Samples were cut using a water-cooled diamond disc to prevent heating of the sample. The resulting sections were fixed in phenolic fiberglass resin (PRESI, Eybens, France), giving them a form that allowed further preparation. The inlaid cross sections were sanded using sandpaper with grain thicknesses ranging from P240 to P1200. Subsequent changes in sandpaper gradation were made after the visible marks were completely ground off. During grinding, the samples were cooled with water to prevent them from overheating. The final step was polishing using diamond paste with an average grain size of 3 µm (Metalogist, Warsaw, Poland). Polishing was continued until the grinding marks were removed. Ongoing evaluation of the grinding and polishing process was performed using a NIKON MA200 optical microscope (Nikon, Minato, Tokyo, Japan). After polishing, the metallographic scrap was cleaned using running water and isopropyl alcohol and dried with compressed air.

**Figure 6:**
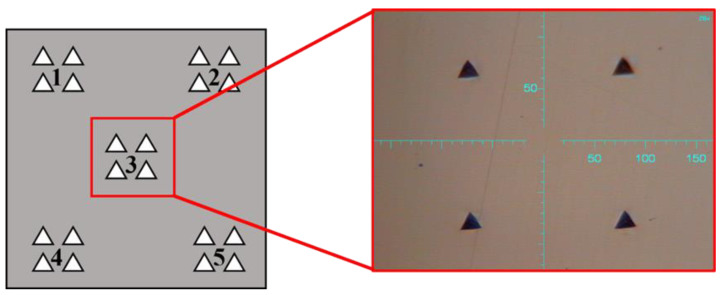
Microhardness measurement scheme and representative indents in the core region of a 316L stainless steel sample produced via SLM technology. Numbers 1–5 indicate measurement areas.

Microhardness measurements were performed with the FISCHERSCOPE HM2000 microhardness tester (Helmut Fischer GmbH, Sindelfingen, Germany), in accordance with the measurement pattern illustrated in Figure 6. Chemical composition analysis was carried out using the PHENOM PRO scanning electron microscope (Thermo Fisher Inc., Waltham, MA, USA), with a magnification range of 160–350,000× and a resolution of 6 nm.

### 2.4. Workspace Orientation and Support Structures

Due to the dimensions and geometries of the compact heat exchanger being fabricated, its orientation in the working space proved to be an important issue. Due to its size (i.e., height exceeding the available working space), the exchanger was oriented at an angle of 15° to the plane of the working platform. This angle was chosen to ensure it did not interfere with the fabrication of the internal channels and to minimize the surface roughness associated with the stepping effect [53].

**Figure 7:**
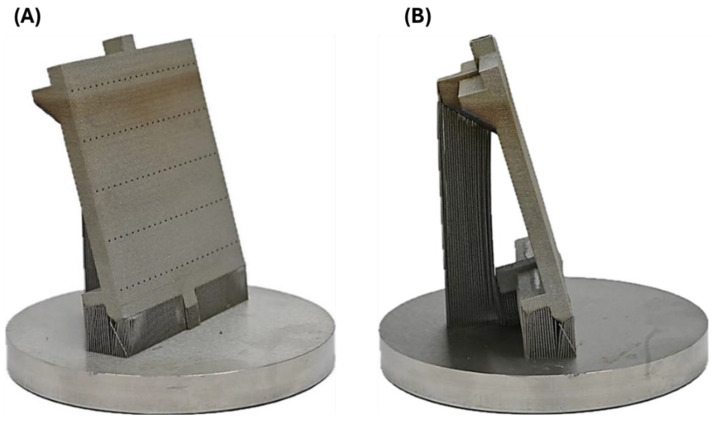
Manufactured compact heat exchanger with a system of support elements: (**A**) front view; (**B**) side view.

Figure 7A shows the front view of the fabricated compact heat exchanger, while Figure 7B illustrates the side view with visible support elements. It is important to note that in the area of internal channels, it was not possible to use supports due to the difficulty of removing them. The lack of support of the fabricated surfaces at an angle to the working platform leads to partial or complete melting of the loose powder grains below the printed layer by the molten material pool, which promotes deformation of the fabricated component and increases surface roughness (adhesion of partially melted powder grains).

The supports play an important role in transferring heat from the print to the powder bed to prevent warping and/or collapse [54]. A significant problem with SLM technology is the thermal stresses created in the print. These can cause deformation of parts and lead to delamination and detachment of supports from the working platform. This problem is compounded for long prints with relatively thin walls [55].

The fabricated compact heat exchanger used a limited set of support structures, which were mechanically removed. The supports provided a stable connection to the working platform. The geometry of the exchanger was consistent with the CAD model. No macrodefects, in the form of cracks and delamination, were observed during visual evaluation. However, some of the surface was slightly deformed, which was analyzed in detail by CT scanning. Additive manufacturing using SLM technology is characterized by high dimensional and shape accuracy. Measurement using electronic calipers showed no significant differences between actual and design dimensions.

**Figure 8:**
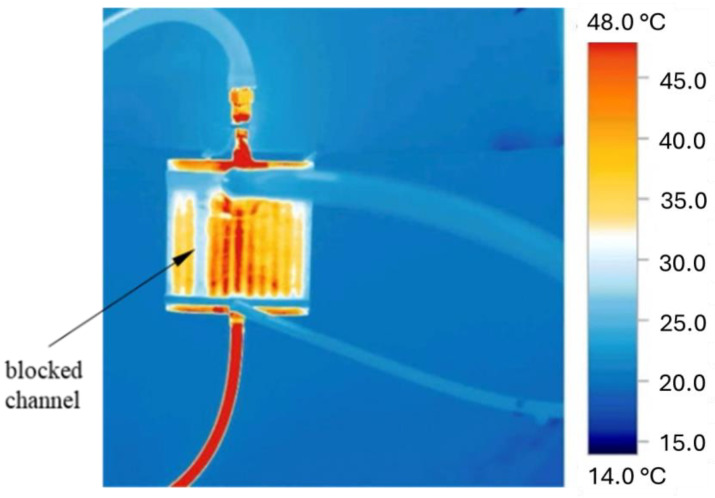
Initial inspection of channel patency using thermal imaging.

After the supports were fabricated and mechanically removed, the exchanger was cleaned ultrasonically in distilled water and with compressed air to remove trapped loose powder grains and other impurities. An initial inspection of channel patency was carried out using a TESTO 890-2 thermal imaging camera (Testo, Titisee-Neustadt, Germany) (Figure 8). The cleaning process was continued until the internal channels were fully cleared, which was subsequently confirmed by CT scans.

### 2.5. Optical Microscopy

In order to analyze structural defects, metallographic studies were performed using a NIKON MA200 optical microscope (Nikon, Minato, Tokyo, Japan) on resin-included sections of the exchanger.

Smaller sections of the exchanger were manufactured additively according to the previously described methodology,.

### 2.6. Contact Profilometry

**Figure 9:**
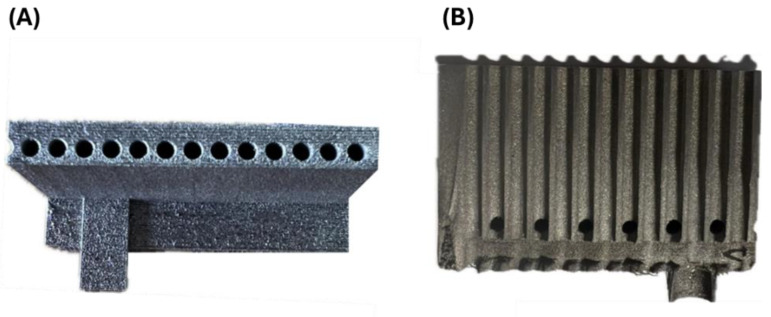
Preparation of the exchanger section for measuring the roughness of the internal channels: (**A**) view before and (**B**) after water jet cutting.

From the point of view of heat transfer, the surface roughness of the internal channels is an important parameter. Due to the physical difficulty in measuring it, a section of the exchanger (Figure 9A) was cut using a water-abrasive jet, as shown in Figure 9B. The roughness measurement was performed using a HOMMEL Tester T8000-R60 contact profilometer (HOMMEL ETAMIC, Villingen-Schwenningen, Germany) with a TK100 sensor on a measuring section of 15 mm in length, with a feed rate of 0.5 mm s^−1^. Due to the size of the prepared sample and the number of channels to be tested (12), the measuring section is shorter than prescribed by ISO 21920-3:2022 [56].

### 2.7. Computed Tomography

Analysis of fabrication accuracy, including structural defects, deformation, and patency of internal channels, was performed using a Phoenix V Tome xS CT scanner (Waygate Technologies, Hürth, Germany). The system includes a 16-bit grayscale detector with a resolution of 1000 × 1000 pixels and a pixel size of 200 μm, a rotary table, and a microfocus X-ray tube with a maximum accelerating voltage of 240 kV. Data analysis was performed with ZEISS INSPECT X-ray 2023 software (ZEISS, Oberkochen, Germany).

## 3. Results and Discussion

### 3.1. Material Characterization

Given the unique characteristics of the additive manufacturing process, material characterization plays a crucial role. One of the most important parameters is relative density, as it directly affects the mechanical properties of the fabricated components. Measurements using a hydrostatic balance demonstrated that the process parameters applied in the fabrication of the heat exchanger resulted in a material relative density of 99.5%.

The next step in the material analysis was the examination of its chemical composition. During the SLM process, evaporation of alloying elements can occur due to the high temperature of the molten pool, which may lead to a chemical composition in the fabricated material that differs from that of the original powder. Table 3 presents the chemical composition measured in a randomly selected area of the cube cross-section. The results show that the chemical composition of the final material differed only marginally from that of the feedstock material MetcoAdd™ 316L-A powder (OerliconMetco Inc., Troy, MI, USA).

Table 4 presents the microhardness measurements of different regions of the metallographic cross-section of the cubic sample. The average microhardness in the core region of the samples fabricated using SLM technology was 255 HV, which is consistent with values reported in the literature [57].

### 3.2. Structural Defects Analysis

**Figure 10:**
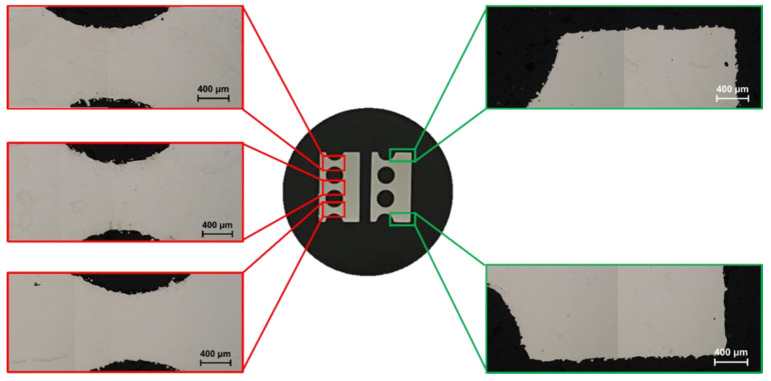
Metallographic images of cross sections of a section of a compact heat exchanger fabricated from 316L stainless steel using SLM technology.

Figure 10 shows photos of finished metallographic scrap sections of a compact heat exchanger. The photos show the compact structure of the material. The absence of structural defects, especially of the “lack of fusion” type, indicates that the process parameters used provided stable conditions for melting and compacting the powder [36,58,59,60,61]. The presence of structural defects, especially those located in near-surface areas, would have a negative impact on the tightness of the exchanger. Flowing fluids could, via the pore system and structural defects, penetrate the walls of the minichannels and mix with each other. In addition, metallographic images from the edge area of the fabricated exchanger show partially melted powder grains, which have a significant impact on the surface roughness.

### 3.3. Surface Roughness of Internal Channels

The surface roughness of additively manufactured parts is mainly influenced by the properties of the construction material [29] (including the morphology and grain size of the powder), the direction of construction [16], and the orientation of the surface in the workspace (angle of inclination with respect to the working platform) [31].

Surface roughness increases with increasing layer height and inclination angle [53]. Decreasing the layer height can reduce the roughness of inclined surfaces, but it will increase the production time and thus the cost of making the part.

**Figure 11:**
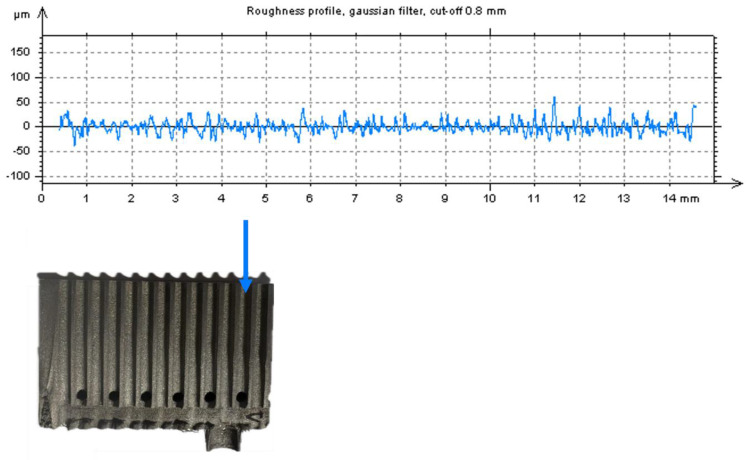
Example of roughness profile for minichannel compact heat exchanger.

Table 5 shows the analyzed roughness parameters obtained from roughness profiles for 12 minichannels of the compact heat exchanger, and Figure 11 shows an example of a roughness profile for one of them. The average value of the Ra parameter for all minichannels was 11.11± 1.63 μm, while the Rz parameter was 74.98 ± 11.13 μm. Similar roughness in their SLM-made exchanger was obtained by Silva et al. [62].

The additively manufactured surface is characterized by high surface roughness, so it is recommended to undergo finishing. Conventional methods such as grinding, polishing, or sandblasting are hindered when the surfaces are curved or inside the fabricated part (e.g., internal channels). Therefore, the search has begun for unconventional and hybrid methods, such as magnetic-abrasive finishing, abrasive flow machining, fluidized bed machining, cavitation-abrasive finishing, or electrochemical machining [63,64].

### 3.4. Analysis of the Accuracy of Execution and Patency of Internal Channels

**Figure 12:**
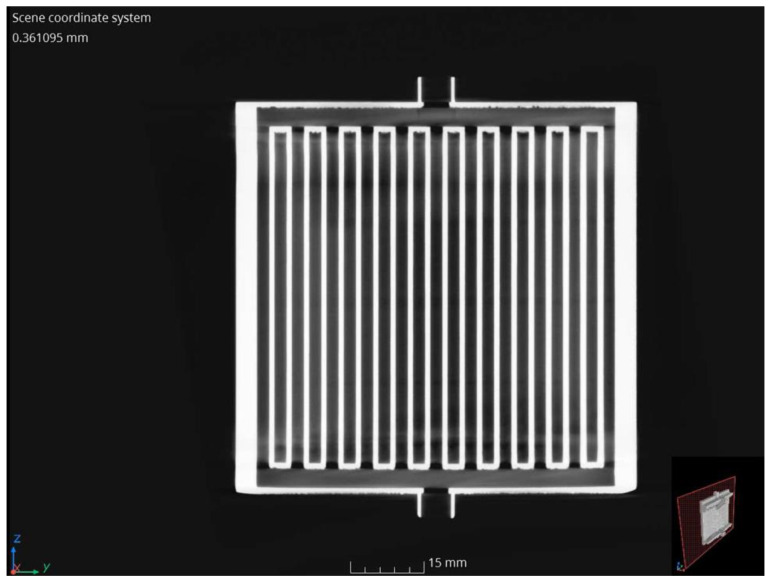
Longitudinal cross-section of the compact heat exchanger with visible arrangement of internal minichannels.

Examination using CT scans allowed analysis of structural defects, deformation, and permeability of internal channels. Analyzing the scans, it can be seen that the geometry of the exchanger was fully mapped. The presence of defects (cracks and discontinuities) in the wall area was not noticed. Figure 12 shows a longitudinal cross-section of the compact heat exchanger with the internal minichannel system visible. Analyzing the photo, the permeability of the minichannels was confirmed, and no loose powder grains were observed.

**Figure 13:**
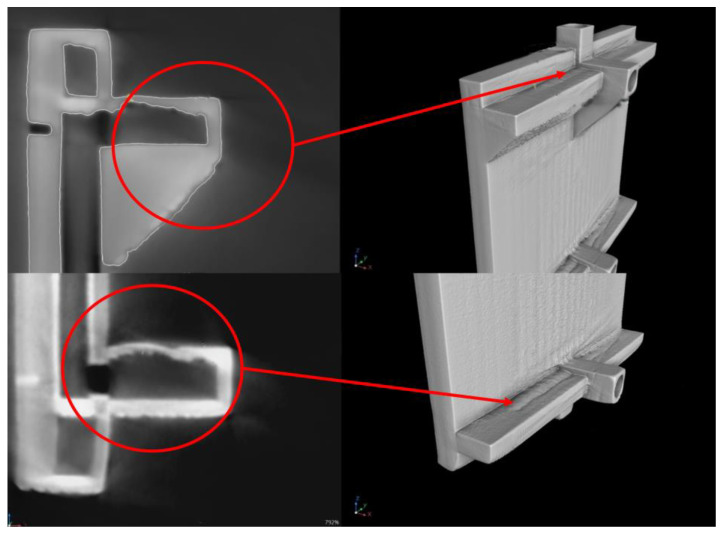
Side cross-section of the compact heat exchanger with visible arrangement of internal minichannels.

Figure 13 shows side cross sections of the compact heat exchanger, which show areas of deformed surfaces located in its upper and lower parts. The probable cause of the deformation was the lack of support of the manufactured surface, which was associated with the formation of a layer atop loose, unmelted powder. Thus, the melting of the powder was disrupted, leading to a bulging and thinner wall thickness in this area than the exchanger’s design indicated. Based on the CT scans, it can be concluded that the observed deformation does not affect the tightness of the exchanger, so it retains its functionality. However, in the future, measures should be taken to prevent deformation.

**Figure 14:**
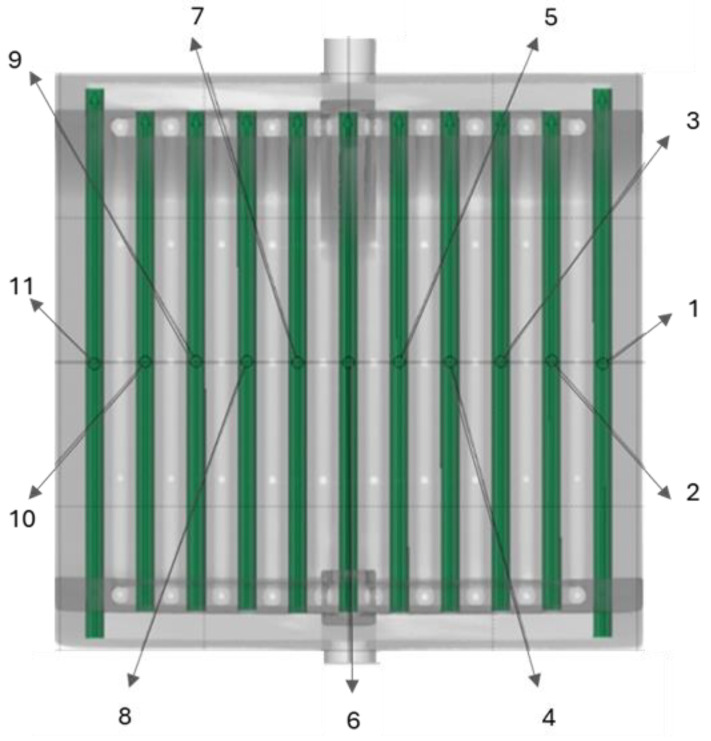
Analysis of the actual hydraulic diameter of the minichannels of the compact heat exchanger—part 1. Numbers 1–11 correspond to the numbering of individual minichannels.

**Figure 15:**
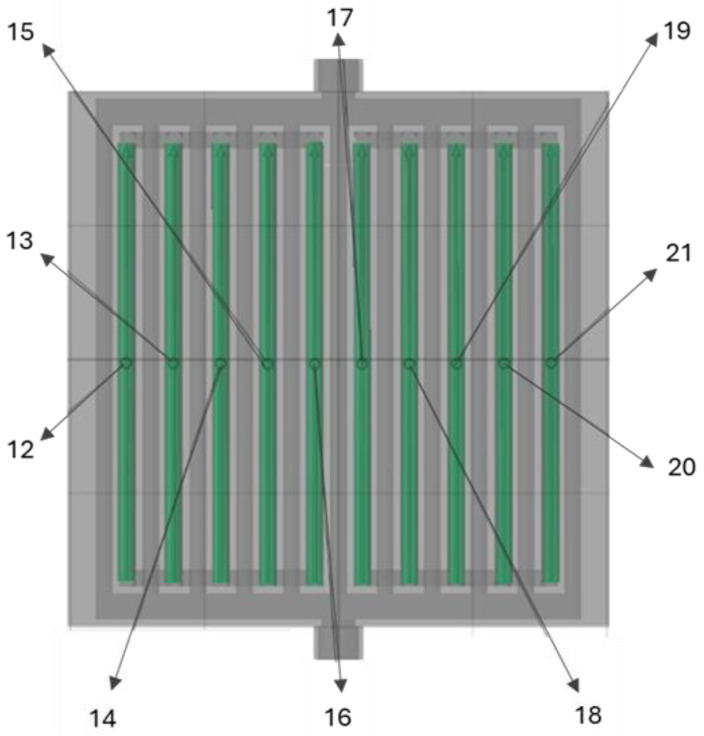
Analysis of the actual hydraulic diameter of the minichannels of the compact heat exchanger, part 2. Numbers 12–21 correspond to the numbering of individual minichannels.

Based on the CT scans, an analysis of the actual hydraulic diameter of the minichannels of the compact heat exchanger was also performed, comparing the actual dimensions with the diameter of a cylinder with a preset dimension of 2.5 mm (the designed hydraulic diameter). The measurement methodology is shown in Figure 14 and Figure 15. The results are summarized in Table 6.

The causes should be attributed to the presence of thin walls between the channels. Thin walls are particularly sensitive to disturbances in powder spreading by the recoater. This results in local material deficits, which exacerbate dimensional deviations. If the distance between the scanned lines (hatch spacing) is too large relative to the wall thickness, part of the powder may remain unmolten. This leads to a reduction in the actual wall thickness compared to the designed value. The accuracy of feature reproduction, especially at the edges, is also influenced by the resolution, which depends on the laser spot size (typically 50–100 µm), the precision of beam guidance, and the part orientation in the build chamber (the stair-stepping effect on inclined surfaces).

## 4. Conclusions

The main objective of the work was to design and verify the feasibility of fabricating a minichannel compact heat exchanger using selective laser melting technology from 316L steel powder. Computed tomography tests confirmed a high level of geometrical accuracy and successful reproduction of the designed geometry.

Due to the orientation of the element in the workspace and the lack of internal supports, part of the surface was slightly deformed. However, this did not affect the functionality of the exchanger.

From the perspective of heat transfer, the surface roughness of the minichannels is an important parameter. The surface roughness and accuracy of the component can be controlled by its orientation in the workspace and by modifying the printing parameters.

The research has shown that when designing a compact heat exchanger based on minichannels to be made using additive technology, special attention should be paid to

- Diameter of the minichannels (to prevent clogging due to powder adhesion),
- Minimum wall thickness (the smaller the wall thickness, the greater the possibility of structural defects and wall discontinuities, which will cause the exchanger to leak),
- Construction angles that will be possible with this technology.

Additive manufacturing, and SLM technology in particular, offers unique opportunities to fabricate complex, lightweight, and highly integrated heat exchanger geometries that are otherwise difficult or impossible to produce. Nevertheless, challenges remain in ensuring repeatability, dimensional precision, and internal surface quality.

The presented exchanger is part of a broader research initiative focused on developing compact heat exchangers for applications with limited space, high heat fluxes, and the need for miniaturization. Although this article focuses on manufacturing and geometric analysis, the design directly addresses the growing need for efficient thermal energy management.

This work links heat transfer enhancement with advanced manufacturing methods and contributes to the development of new design methodologies. The results are relevant to multiple industries, including HVAC, automotive and transportation, electronics, and medical devices. Future research will focus on experimental evaluation of condensation processes under two-phase flow conditions in such exchangers.

## Figures and Tables

**Table 1:** Chemical composition of MetcoAdd^TM^ powder [52].

Element	Fe	Cr	Ni	Mo	C	Other
Percentage by weight [%]	65	18	12	2	<0.03	<1

**Table 2:** Parameters of the selective laser melting process.

Laser Power [W]	Scanning Speed [mm s^−1^]	Layer Height [mm]	Hatch Distance [mm]	Protective Atmosphere
250	900	0.025	0.06	Argon

**Table 3:** Chemical composition of the heat exchanger material.

Element	Fe	Cr	Ni	Mo	C	Other
Percentage by weight [%]	60	17	12	1.5	<0.03	<1

**Table 4:** Results of HV0.05 microhardness measurements for the 316L stainless steel sample fabricated using SLM technology.

Part	Measurement Number HV_0.05_				Average HV_0.05_
	P1	P2	P3	P4	
1	251	264	260	274	262 ± 8
2	244	243	251	239	244 ± 5
3	259	241	255	263	255 ± 8
4	270	285	272	245	268 ± 14
5	244	240	234	236	238 ± 4

**Table 5:** Roughness parameter values determined from roughness profiles for minichannels.

Ra [μm]	10.40	11.02	11.20	12.41	13.67	9.23	11.11 ± 1.63
	12.77	9.88	8.89	8.70	12.40	12.83	
Rz [μm]	63.42	79.68	74.90	82.80	86.61	65.09	74.98 ± 11.13
	95.72	67.20	60.88	59.62	79.15	84.64	

**Table 6:** Summary of the actual diameter of the minichannels of the compact heat exchanger.

Channel Number	Actual Diameter [mm]	Deviation [mm]	Channel Number	Actual Diameter [mm]	Deviation [mm]	Channel Number	Actual Diameter [mm]	Deviation [mm]
1	2.6036	0.1036	8	2.5789	0.0789	15	2.5848	0.0848
2	2.6016	0.1016	9	2.5961	0.0961	16	2.5795	0.0795
3	2.5986	0.0986	10	2.5863	0.0863	17	2.5858	0.0858
4	2.5907	0.0907	11	2.5835	0.0835	18	2.5867	0.0867
5	2.5888	0.0888	12	2.5890	0.0890	19	2.5997	0.0997
6	2.5860	0.0860	13	2.5883	0.0883	20	2.6031	0.1031
7	2.5856	0.0856	14	2.5938	0.0938	21	2.6095	0.1095

## Data Availability

The original contributions presented in this study are included in the article. Further inquiries can be directed to the corresponding authors.
